# Maternal Use of Selective Serotonin Reuptake Inhibitors and Lengthening of the Umbilical Cord: Indirect Evidence of Increased Foetal Activity—A Retrospective Cohort Study

**DOI:** 10.1371/journal.pone.0154628

**Published:** 2016-04-29

**Authors:** Julia Kivistö, Soili M. Lehto, Katja Halonen, Leena Georgiadis, Seppo Heinonen

**Affiliations:** 1 Department of Psychiatry, Institute of Clinical Medicine, University of Eastern Finland, Kuopio, Finland; 2 Department of Psychiatry, Kuopio University Hospital, Kuopio, Finland; 3 Department of Pharmacology and Toxicology, University of Eastern Finland, Kuopio, Finland; 4 Department of Obstetrics and Gynaecology, Institute of Clinical Medicine, University of Eastern Finland, Kuopio, Finland; 5 Department of Obstetrics and Gynaecology, Helsinki University Hospital, Helsinki, Finland; 6 Department of Obstetrics and Gynaecology, University of Helsinki, Helsinki, Finland; Radboud University Medical Centre, NETHERLANDS

## Abstract

**Background:**

Antenatal depression affects up to 19% of pregnant women. Some of these women are also in need of antidepressant treatment. Nevertheless, the impact of maternal antidepressant treatment and prenatal depression on the course of pregnancy, foetal development and delivery outcomes is not fully understood.

**Methods:**

We analysed data from 24 818 women who gave birth at Kuopio University Hospital between 2002–2012. Logistic regression analysis was used to estimate associations between the use of selective serotonin reuptake inhibitors (SSRIs) during pregnancy and the progression of pregnancy, development of the foetus and delivery outcomes.

**Results:**

Altogether, 369 (1.5%) women used SSRIs. A regression model adjusted for age, overweight, nulliparity, prior termination, miscarriages, smoking, maternal alcohol consumption, chronic illness and polyhydramnion showed that pregnant women exposed to SSRI medication had significantly lower Apgar scores at 1 minute (p < 0.0001) and 5 minutes (p < 0.0001) and more admissions to the neonatal intensive care unit (p < 0.0001) than unexposed pregnant women. In addition, exposed newborns had longer umbilical cords (p < 0.0001) than non-exposed newborns.

**Conclusion:**

In addition to the previously known associates with maternal SSRI exposure, such as lowered Apgar scores, SSRI exposure appeared to be associated with increased umbilical cord length. The observation related to increased umbilical cord length may be explained by an SSRI-induced increase in the movements of the developing foetus.

## Introduction

Antenatal depression is a common condition that affects approximately 7–19% of pregnant women [1, 2]. Of these women, approximately 7–9% have been estimated to use antidepressant medication during the prenatal period [3, 4]. Selective serotonin reuptake inhibitors (SSRI) are considered relatively safe for treating psychiatric conditions during the antenatal period [5]. Nevertheless, increased risks for some maternal and foetal conditions, such as spontaneous miscarriages and low birth weight, have been reported [6, 7].

In the past several years, the use of SSRI medication during the antenatal period has substantially increased; SSRIs are nowadays the most frequently used antidepressant medication for depressive disorders generally, and also during pregnancy [4]. Antenatal SSRI use may lead to pregnancy complications such as spontaneous miscarriages and preeclampsia [7–9]. Maternal SSRI medication has also been associated with poorer delivery outcomes such as preterm birth, low birth weight, birthweight low for gestational age (SGA) and low Apgar scores [6, 10 –14]. In addition, SSRI medication during the antenatal period has been linked to neonatal adaptation difficulties and shortness of breath in the offspring [15, 16]. Newborns of pregnant women exposed to SSRI medication also had more neonate intensive care unit admissions than unaffected mothers [11, 15]. Nevertheless, findings regarding antenatal SSRI drug use and its effects on the progression of pregnancy, delivery outcomes and the cognitive and emotional development of the child are somewhat conflicting [5, 6, 13]. Such discrepancies may be explained by differences in the investigated populations, such as varying criteria for sample selection. Furthermore, a recent meta-analysis [5] concluded that a sufficient amount of direct evidence was only available to evaluate antidepressant effects on preterm birth, neonatal convulsions and respiratory distress. In the same meta-analysis, only the likelihood of respiratory distress was elevated in mothers using antidepressant medications.

The aim of this retrospective cohort study, utilizing a large naturalistic cohort collected during ten consecutive years, was to investigate the effects of SSRI exposure on the progress of pregnancy, development of the foetus and delivery outcomes in one single unit using uniform diagnostic criteria and treatment policies.

## Materials and Methods

### 2.1 Study setting and subjects

We analysed data from 24 818 women who gave birth at Kuopio University Hospital, located in Eastern Finland, between 2002–2012. Kuopio University Hospital is a tertiary level perinatal centre in Eastern Finland and also the only delivery hospital in the North Savo district, and the data therefore covered the entire population of women giving birth in the area. The data were gathered retrospectively from the hospital birth register. All the utilized data were anonymous and contained no personal information. Therefore, in accordance with Finnish legislation, no informed consent was required. The Ethics Committee of the Kuopio University Hospital approved the study. (Reference number 93/2008).

### 2.2 Background variables

The hospital register data were originally collected using a questionnaire. First, the participants filled in the background information section of the questionnaire. The rest of the questionnaire was completed by a midwife during interviews conducted at antenatal maternity health care visits and at delivery in Kuopio University Hospital.

In the questionnaire, the participants reported the use of contraceptives (yes/no), regular smoking (yes/no), alcohol consumption (yes/no) and possible chronic illnesses (yes/no). The chronic illnesses comprised arterial hypertension, rheumatoid arthritis and other systemic connective tissue diseases, heart defects, asthma, kidney diseases, epilepsy and thyroid diseases. The numbers of previous caesarean sections, labours and terminations were reported by the participants and the information was completed by midwife interviews. The birth register included information regarding delivery outcomes. In addition, information on the newborns from immediately after birth until the age of seven days was available for all pregnancies lasting more than 22 gestational weeks.

### 2.3 Measurements

After each delivery, the placenta was routinely examined by trained nursing staff. Placental weight was measured in grams with the umbilical cord and membranes attached. The insertion and the length of the umbilical cord were examined and measured in a validated way, as previously described [17]. The umbilical cord length was measured and expressed as a standard deviation (SD) from the gestational age- and sex-specific mean values according to growth curve measurements, as previously described [17]. Diagnoses of velamentous umbilical cord insertion and placental abruption were set clinically or by ultrasound examination by the delivery team. The amniotic fluid volume was assessed by trained nursing staff during the delivery and classified into three groups: the normal, oligo- or polyhydramnion group. The amniotic fluid volume was not routinely examined using the ultrasound procedure.

Body mass index (BMI) was calculated by dividing the body weight in kilograms by the squared height in metres. The diagnosis of GDM was based on the contemporary criteria of oral glucose tolerance test (OGTT): fasting blood glucose > 4.8 mmol/l, 1-hour blood glucose > 10.0 mmol/l and 2-hour blood glucose > 8.7 mmol/l until September 2001, and since September 2001, fasting plasma glucose (FPG) > 4.8 mmol/l, 1-hour plasma glucose (PG) > 11.2 mmol/l and 2-hour plasma glucose > 9.9 mmol/l. One or more elevated values during an OGTT resulted in the diagnosis of GDM [18]. The criteria of the American College of Obstetricians and Gynecologists (ACOG) were used to define preeclampsia: systolic blood pressure ≥ 140 mmHg or diastolic blood pressure ≥ 90 mmHg in two separate measurements at least 6 h apart after 20 weeks of gestation in a previously normotensive woman with a 24-h urinary protein excretion of ≥ 0.3 g [19]. Maternal anaemia was defined as haemoglobin levels below 120 g/l. Amnionitis diagnosis was based on the ICD-10 code and clinically set by obstetricians at the time of delivery. Birth was considered preterm when the infant was delivered before gestational week 37. Low birth weight was defined as a birth weight of less than 2500 g. Newborns were considered small for gestational age (SGA) when the sex- and age-adjusted birth weight was below the tenth percentile, according to the Finnish standards [20]. Low Apgar scores were defined as score of less than 7 at 1 or 5 minutes. In the case of several abnormalities at the same time, such as preterm birth and a small size for the gestational age, all the recorded abnormalities were considered as independent variables.

### 2.4 Antidepressant use

Altogether, 1.7% of all pregnant women in this study (n = 24 818) used antidepressant preparations (n = 416). The most common antidepressant group was SSRIs (n = 369). Eleven women were excluded from the SSRI group due to combined therapy with an SSRI and an antidepressant belonging to another pharmacological group. Therefore, the study group consisted of 358 women using only antidepressants belonging to the SSRI group during pregnancy. The SSRIs used, ranked according to the frequency of use, were citalopram (n = 217), sertraline (n = 56), fluoxetine (n = 54), paroxetine (n = 34) and fluvoxamine (n = 4). Seven women from the SSRI group (n = 358) used two different SSRI antidepressants at the same time during the pregnancy. In the drug-specific analysis, we excluded seven mothers due to combined SSRI therapy with two different SSRI antidepressants. In addition, the fluvoxamine group (n = 4) was excluded due to the small group size. Therefore, the SSRIs used in the drug-specific data included citalopram (n = 215), sertraline (n = 49), fluoxetine (n = 51) and paroxetine (n = 32). Women using antidepressant agents belonging to pharmacological groups other than SSRIs were excluded due to the small group sizes precluding sufficiently powered analyses of preparations belonging to specific antidepressant classes (tricyclic antidepressants: n = 41; venlafaxine: n = 23; mirtazapine: n = 13; trazodone: n = 1). The control group (n = 24 402) consisted of the pregnant women not using any antidepressant medication.

### 2.5 Statistical analysis

Data were analysed using SPSS 19.00 for Windows. Differences between women using SSRI medication (n = 358) and those not using antidepressants (n = 24 402) were assessed using the χ^2^ test and Fisher’s exact test for dichotomous variables. Continuous variables were analysed using the Student’s t-test. P values <0.05 were considered significant.

Multivariate analysis was conducted with logistic regression (dependent variable: exposure to SSRIs vs. no exposure) and adjusted for all mother-related factors and risks related to pregnancy and labour that were statistically significant in the unadjusted group difference analyses. In this study, the variables included in the multivariate analyses were mother’s age > 35 y (yes/no), pre-gravid BMI > 26 (yes/no), nulliparity (yes/no), prior miscarriage (yes/no), prior termination (yes/no), chronic illness (yes/no), gestational diabetes (yes/no), maternal alcohol consumption (yes/no), maternal smoking > 5 cigarettes per day (yes/no) and polyhydramnion (yes/no). The multivariate analyses were separately conducted for each delivery outcome: (1) preterm delivery, 2) caesarean, 3) low (<7) Apgar score at 1 min, 4) low (<7) Apgar score at 5 min, 5) neonatal intensive care, 6) vacuum assistance, 7) perinatal mortality, 8) low birth weight (<2500 g), 9) SGA (<10th centile), 10) LGA (>10th centile), 11) umbilical cord length <5^th^ centile, 12) umbilical cord length <10^th^ centile, 13) umbilical cord length >90^th^ centile, 14) umbilical cord length >95^th^ centile, 15) head circumference <10^th^ centile, 16) head circumference >90^th^ centile, 17) length of the child <10^th^ centile, and 18) length of the child >90^th^ centile.

## Results

### 3.1 Maternal Risk factors

Distributions of the maternal risk factors are presented in Table 1. Pregnant women exposed to SSRI medication had a higher pre-gravid BMI, used alcohol and smoked during pregnancy more often than women not exposed to SSRIs. In addition, the SSRI group had greater rates of terminations, miscarriages and chronic illnesses. Moreover, nulliparity was more common in the SSRI group.

**Table 1:** Maternal Risk Factors and pregnancy characteristics.

Maternal risk factors	Any SSRI (n = 358) (n) %	No SSRI (n = 24 402) (n) %	*p*-value
Age (y)>35	(58) 16	(3338) 13.7	**0.01**^**2**^
<18	(1) 0.3	(149) 0.6	**0.64**^**1**^
Pregravid BMI > 26 kg/m^2^	(118) 34	(6537) 28.2	**0.01**^**2**^
Smoking (>5 cigarettes /day)	(48) 13.4	(1003) 4.1	**<0.0001**^**2**^
Pregravid alcohol consumption (yes)	(126) 36.7	(9479) 40.7	**0.08**^**2**^
Alcohol consumption during pregnancy (yes)	(10) 2.9	(137) 0.6	**<0.0001**
Chronic illness (yes)^3^	(63) 17.6	(3403) 13.9	**0.03**^**2**^
Diabetes (yes)	(3) 0.8	(223) 0.9	**1.00**^**2**^
Hypertension (yes)	(8) 2.2	(502) 2.1	**0.55**^**1**^
Low haemoglobin (<120 g/l)	(12) 3.4	(670) 2.7	**0.48**^**1**^
Nulliparity (yes)	(116) 32.4	(9955) 40.8	**0.001**^**2**^
Prior termination (yes)	(62) 17.3	(3042) 12.5	**0.007**^**1**^
Prior caesarean section (yes)	(35) 9.8	(1785) 7.3	**0.08**^**1**^
Prior miscarriage (yes)	(95) 26.5	(4839) 19.8	**0.002**^**1**^

^1^ χ^2^ test

^2^ Fisher’s exact test

BMI = body mass index

^3^Arterial hypertension, rheumatoid arthritis and other systemic connective tissue diseases, heart defects, asthma, kidney diseases, epilepsy and thyroid diseases

Information lacking from the data: breech presentation (n = 734), velamentous umbilical cord insertion (n = 740)

### 3.2. Pregnancy and delivery characteristics

Pregnancy and delivery related variables are presented in Table 2. With regard to pregnancy characteristics, gestational diabetes (p = 0.002) and polyhydramnion (p = 0.04) were more common in the SSRI group compared with women not exposed to SSRIs. The duration of pregnancy was significantly shorter in women exposed to SSRIs (p = 0.02). Moreover, women exposed to SSRIs displayed more hospital care days after delivery (p < 0.0001), a higher placental weight (p = 0.03), longer umbilical cords (p < 0.0001) and a smaller newborn head circumference (p = 0.01) than women not exposed to SSRIs.

**Table 2:** Pregnancy and delivery characteristics.

Result	SSRI use(n = 358) (n) %	No SSRI use(n = 24 402) (n) %	*p*- value
Variable, n (%)			
Gestational diabetes (yes)	(73) 20.4	(3606) 14.8	**0.002**^**1**^
Amnionitis (yes)	(0.0) 0	(121) 0.5	**0.43**^**2**^
Placenta praevia (yes)	(4) 1.1	(201) 0.8	**0.55**^**2**^
Pre-eclampsia (yes)	(19) 5.3	(1235) 5.1	**0.82**^**1**^
Polyhydramnion (yes)	(23) 6.4	(1002) 4.1	**0.04**^**1**^
Oligohydramnion (yes)	(44) 12.3	(2850) 11.7	**0.69**^**2**^
Placental abruption (yes)	(1) 0.3	(173) 0.7	**0.52**^**2**^
Breech presentation (yes)	(25) 7.0	(1309) 5.5	**0.24**^**1**^
Foetal gender (male)	(186) 52.8	(12581) 51.6	**0.15**^**2**^
Velamentous umbilical cord insertion (yes)	(14) 4.0	(649) 2.7	**0.27**^**2**^
Variable, mean ± SD			
Duration of pregnancy (days)	247.0 ± 14.7	276.0 ± 16.6	**0.02**^**3**^
Duration of hospital care after delivery (days)	3.9 ± 2.2	3.3 ± 1.9	**<0.0001**^**3**^
Increase in weight during pregnancy (kg)	13.4 ± 5.3	13.9 ± 5.3	**0.18**^**3**^
Pregravid BMI (kg/m^2^)	24.9 ± 5.4	24.1 ± 16.8	**0.42**^**3**^
BMI at the end of pregnancy (kg/m^2^)	30.0 ± 5.2	29.3 ± 23.9	**0.69**^**3**^
Placental weight (g)	639.0 ± 152.2	616.0 ± 191.9	**0.03**^**3**^
Head circumference (cm)	34.7 ± 1.8	35.0 ± 2.2	**0.01**^**3**^
Umbilical cord length (cm)	62.2 ± 14.9	59.30 ± 13.9	**<0.0001**^**3**^
Body circumference (cm)	34.0 ± 2.1	34.0 ± 2.2	**0.97**^**3**^
Birth weight (g)	3387.1 ± 649.3	3436.5 ± 642.9	**0.15**^**3**^

^1^ χ^2^ test

^2^ Fisher’s exact test

^**3**^ Student’s t-test

BMI = body mass index

SD = standard deviation

Information lacking from the data: Placental weight (n = 311), head circumference (n = 722), umbilical cord length (n = 318), body circumference (n = 12618), birth length (n = 7095), duration of pregnancy (n = 25), weight gain during pregnancy (n = 85)

### 3.3. Multivariate analysis of pregnancy and delivery outcomes

After adjustments for maternal age, pregravid BMI, nulliparity, previous terminations, previous miscarriages, smoking, alcohol consumption, chronic illness, gestational diabetes and polyhydramnion, having low Apgar scores (<7) at 1 and 5 minutes was also associated with an increased likelihood of belonging to the SSRI group at an odds ratio of 2.3 at 1 minute (p < 0.0001) and 2.3 at the age of 5 minutes (p < 0.0001) (Table 3). Moreover, being admitted to the neonatal intensive care unit was associated with a 2.2-fold increased likelihood of belonging to the SSRI group (p < 0.0001).

**Table 3:** Multivariate analyses regarding delivery characteristics.

Explanatory variable	SSRI use(n = 358)(n) %	No SSRI use(n = 24 402)(n) %	OR95% CI	Adjusted^1^ OR 95% CI	*p*-value^1^
Preterm delivery^**2**^ (yes)	(39) 10.9	(1909) 7.8	1.44 (1.03–2.02)	1.35 (0.95–1.93)	0.10
Caesarean (yes)	(73) 2.0	(4216) 17.3	1.20 (0.93–1.55)	1.15 (0.88–1.51)	0.30
Low (<7) Apgar score at 1 min	(51) 12.3	(1451) 5.9	2.27 (1.66–3.12)	2.29 (1.65–3.18)	<0.0001
Low (<7) Apgar score at 5 min.	(27) 6.5	(780) 3.2	2.37 (1.58–3.56)	2.30 (1.50–3.52)	<0.0001
Neonatal intensive care (yes)	(82) 19.7	(2431) 10.0	2.28 (1.75–2.96)	2.20 (1.67–2.88)	<0.0001
Vacuum assistance (yes)	(23) 8.0	(1609) 8.8	0.90 (0.59–1.38)	1.00 (0.65–1.55)	0.99
Perinatal mortality (yes)	(3) 0.8	(130) 0.5	1.58 (0.50–4.98)	1.94 (0.61–6.19)	0.26
Low birth weight(<2500 g)	(30) 8.4	(1573) 6.4	1.33 (0.91–1.94)	1.22 (0.81–1.82)	0.34
SGA^3^	(29) 8.5	(2336) 10.0	0.84 (0.57–1.23)	0.80 (0.53–1.18)	0.25
LGA^4^	(35) 10.3	(2191) 9.4	1.10 (0.78–1.57)	0.99 (0.69–1.46)	0.99

OR = odds ratio, CI (confidence interval)

^**1**^maternal age over 35, BMI (pregravid) over 26, nulliparity, prior termination, miscarriage, maternal smoking (>5 cigarettes /d), maternal alcohol consumption, chronic illness, gestational diabetes, polyhydramnion

^**2**^ Birth before 37 completed weeks.

^3^ Small for gestational age (< 10th centile) [20]

^4^ Large for gestational age (>10th centile) [20]

**Fig 1:**
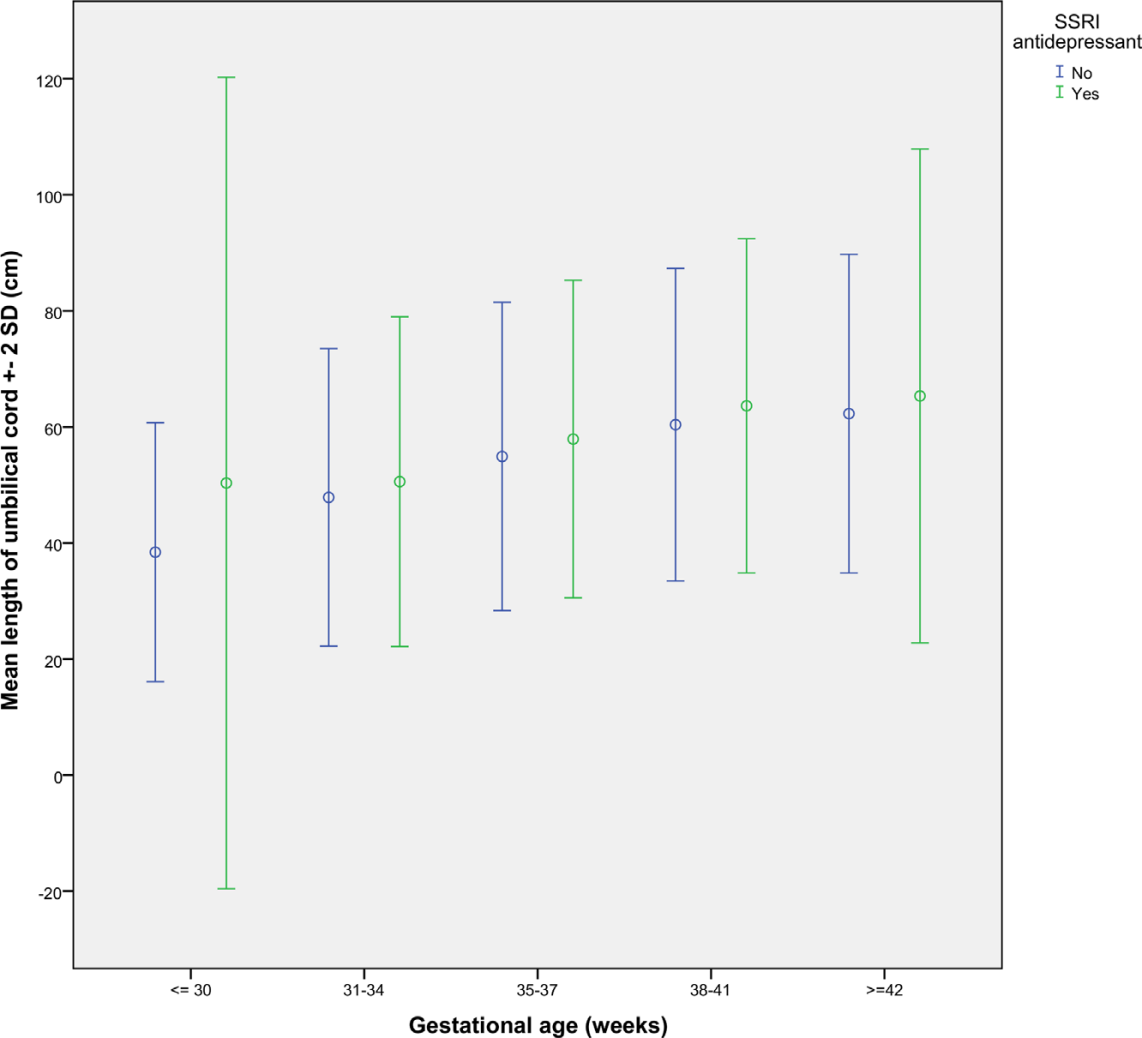
The mean umbilical cord length (± SD) in women with and without SSRI medication during pregnancy at different gestational ages.

Table 4 presents the results of multivariate analyses regarding the delivery outcomes between the study groups. Having an umbilical cord length over the 90th centile was associated with a 1.6-fold, and a length over the 95th centile with a 1.7-fold likelihood of belonging to the SSRI group. Differences in umbilical cord length among women with and without SSRI medication at different gestational ages are illustrated in Fig 1.

**Table 4:** Multivariate analyses regarding delivery outcomes (centile).

Explanatory variable	SSRI use(n = 369)% (n)	No SSRI use(n = 24 402)% (n)	OR95% CI	Adjusted^1^OR95% CI	*p*-value^1^
Umbilical cord length <5^th^ centile	(10) 3.0	(1208) 5.2	0.56 (0.30–1.05)	0.54 (0.28–1.06)	0.07
Umbilical cord length <10^th^ centile	(24) 7.1	(2300) 9.9	0.70 (0.46–1.06)	0.74 (0.48–1.13)	0.16
Umbilical cord length >90^th^ centile	(69) 20.5	(3076) 13.3	1.68 (1.29–2.20)	1.64 (1.24–2.17)	0.001
Umbilical cord length >95^th^ centile	(36) 10.7	(1555) 6.7	1.66 (1.17–2.36)	1.65 (1.15–2.36)	0.007
Head circumference <10^th^ centile	(28) 8.8	(1709) 7.5	1.18 (0.80–1.75)	1.20 (0.80–1.79)	0.38
Head circumference>90^th^ centile	(25) 7.9	(2569) 11.3	0.67 (0.44–1.01)	1.48 (0.97–2.25)	0.07
Length of the child <10^th^ centile	(35) 13.3	(1847) 11.1	1.22 (0.85–1.75)	1.18 (0.81–1.72)	0.39
Length of the child >90^th^ centile	(15) 5.7	(1481) 8.9	0.62 (0.37–1.04)	0.64 (0.38–1.08)	0.09

^**1**^maternal age over 35 y, BMI (pregravid) over 26, nulliparity, prior termination, miscarriage, maternal smoking (>5 cigarettes /d), maternal alcohol consumption, chronic illness, gestational diabetes, polyhydramnion. Information lacking from the data: umbilical cord length centile (n = 1217), head circumference centile (n = 1724), length of the newborn centile (n = 7851)

Table 5 presents the drug-specific data for the four different SSRI antidepressants (fluoxetine, citalopram, paroxetine and sertraline) used in the study. Admission to a neonatal intensive care unit and an umbilical cord length over the 90^th^ centile were associated with all antidepressant types except sertraline.

**Table 5:** Multivariate analyses regarding pregnancy and delivery outcomes among different SSRI antidepressants. All analyses were adjusted for maternal age over 35 y, BMI (pregravid) over 26, nulliparity, prior termination, miscarriage, maternal smoking (>5 cigarettes/d), maternal alcohol consumption, chronic illness, gestational diabetes and polyhydramnion.

Explanatory variable	Fluoxetine (n = 51)	Citalopram (n = 215)	Paroxetine (n = 32)	Sertraline (n = 49)	All SSRIs *p*-value (n = 358)
Preterm delivery^**1**^ (yes)	p = 0.63OR 1.27(0.48–3.32)	p = 0.43OR 1.21(0.75–1.96)	p = 0.14OR 2.11(0.78–5.69)	p = 0.91OR 0.94(0.29–3.06)	0.10
Caesarean (yes)	p = 0.30 OR 1.42(0.73–2.77)	p = 0.98 OR 1.00(0.70–1.46)	p = 0.08 OR 2.02(0.91–4.47)	p = 0.60 OR 1.23(0.58–2.60)	0.30
Low (<7) Apgar score at 1 min	p = 0.04 OR 2.42(1.06–5.54)	p<0.001 OR 2.60(1.73–3.91)	p = 0.36 OR 1.76(0.52–5.98)	p = 0.67 OR 1.30(0.40–4.24)	<0.0001
Low (<7) Apgar score at 5 min.	p = 0.25 OR 2.00(0.62–6.50)	p<0.001 OR 2.61(1.55–4.40)	p = 0.97 OR 1.04(0.14–7.70)	p = 0.03 OR 3.24(1.13–9.23)	<0.0001
Neonatal intensive care (yes)	p = 0.03 OR 2.23(1.09–4.55)	p<0.001 OR 2.12(1.48–3.03)	p = 0.001 OR 3.71(1.66–8.26)	p = 0.74 OR 1.17(0.45–3.03)	<0.0001
Vacuum assistance (yes)	p = 0.60 OR1.32(0.46–3.79)	p = 0.61 OR 0.85(0.46–1.59)	p = 0.91 OR 1.09(0.25–4.78)	p = 0.34 OR 1.70(0.58–4.95)	0.99
Perinatal mortality (yes)	p = 1.0	p = 0.05 OR 3.25 (1.01–10.46)	p = 1.0	p = 1.0	0.26
Low birth weight (<2500 g)	p = 0.41 OR 0.53(0.12–2.40)	p = 0.17 OR 1.42(0.86–2.32)	p = 0.81 OR 0.83(0.19–3.67)	p = 0.93 OR 1.06(0.32–3.51)	0.34
SGA^2^	p = 0.80 OR 1.13(0.44–2.92)	p = 0.85 OR 0.95(0.59–1.55)	p = 0.47 OR 0.59(0.14–2.49)	p = 0.15 OR 0.23(0.03–1.68)	0.25
LGA^3^	p = 0.29 OR 0.53(0.16–1.73)	p = 0.81 OR 1.06(0.67–1.69)	p = 0.83 OR 1.14(0.34–3.87)	p = 0.95 OR 1.03(0.36–2.97)	0.99
Umbilical cord length <5^th^ centile	p = 0.85 OR 0.87(0.21–3.61)	p = 0.04 OR 0.30(0.10–0.93)	p = 0.69 OR 1.34(0.32–5.68)	p = 0.87 OR 1.13(0.27–4.72)	0.07
Umbilical cord length <10^th^ centile	p = 0.73 OR 1.18(0.46–3.00)	p = 0.08 OR 0.58(0.31–1.06)	p = 0.97 OR 1.02(0.31–3.40)	p = 0.72 OR 1.21(0.43–3.45)	0.16
Umbilical cord length >90^th^ centile	p = 0.03 OR 2.10(1.08–4.07)	p = 0.01 OR 1.60(1.12–2.28)	p = 0.03 OR 2.49(1.10–5.64)	p = 0.64 OR 0.78(0.27–2.21)	0.001
Umbilical cord length >95^th^ centile	p = 0.15 OR 1.88(0.79–4.46)	p = 0.02 OR 1.71(1.09–2.69)	p = 0.94 OR 1.05(0.25–4.45)	p = 0.34 OR 1.67(0.59–4.76)	0.007
Head circumference <10^th^ centile	p = 0.27 OR 1.70(0.66–4.40)	p = 0.31 OR 1.30(0.78–2.16)	p = 0.87 OR 0.89(0.21–3.80)	p = 0.90 OR 1.08(0.33–3.59)	0.38
Head circumference>90^th^ centile	p = 0.95 OR 0.97(0.38–2.52)	p = 0.03 OR 0.51(0.28–0.93)	p = 0.60 OR 1.33(0.46–3.90)	p = 0.89 OR 0.93(0.33–2.65)	0.07
Length of the child <10^th^ centile	p = 0.15 OR 1.85(0.80–4.27)	p = 0.83 OR 1.06(0.63–1.78)	p = 0.89 OR 1.09(0.32–3.76)	p = 0.26OR 1.68(0.68–4.15)	0.39
Length of the child >90^th^ centile	p = 0.31 OR 0.48(0.11–2.00)	p = 0.53 OR 0.82(0.44–1.53)	p = 0.50OR 0.50(0.07–3.73)	p = 0.25OR 0.31(0.42–2.28)	0.09

OR = odds ratio, CI (confidence interval)

^**1**^ Birth before 37 completed weeks.

^2^ Small for gestational age (<10^th^ centile) [20]

^3^ Large for gestational age (>10^th^ centile) [20]

Information lacking from the data: umbilical cord length centile (n = 1217), head circumference centile (n = 1724), length of the newborn centile (n = 7851)

## Discussion

We observed a novel association between maternal SSRI antidepressant use and an increased length of the umbilical cord in this cohort of more than 24 000 pregnant women. The findings remained significant after adjustments for a number of potential confounding factors. The increase in the length of umbilical cord appeared to be consistent at different gestational stages, suggesting that the finding did not simply occur by chance in pregnancies going to term. In addition, we replicated some of the observations derived from other cohorts, such as an increased likelihood of lower Apgar scores in pregnant women using SSRI medications.

The newborns of mothers exposed to SSRI medication had lower Apgar scores than the controls, which was line with previous observations [10,13]. Moreover, similarly to others [11, 14], we observed that the newborns of pregnant women exposed to SSRIs had more neonatal intensive care unit admissions than the control group. However, we observed no correlation between maternal SSRI use and a low birth weight or a preterm delivery. These observations are in line with some [14, 17, 21], but not all previous studies [6, 7, 10–12, 15, 22].

The association between increased umbilical cord length and maternal SSRI use has not previously been observed. We observed that a longer umbilical cord was associated with belonging to the fluoxetine, citalopram and paroxetine group. Such a finding may be explained by the tendency of SSRI antidepressants to induce increased foetal movement in the uterus, which may in turn lead to lengthening of the umbilical cord. This hypothesis is based on the finding that movements of the foetus stretch the umbilical cord, indicating that hyperactive foetuses have longer umbilical cords than more passive foetuses [23]. The hypothesis is also supported by some previous studies demonstrating that the umbilical cord is longer in pregnancies where the uterine cavity is larger due to advancing parity, GDM or polyhydramnion, or when the birth weight is higher [17,24–27]. In our study, GDM and polyhydramnion were more common among SSRI-medicated mothers; however, adjustments for these factors did not alter the results.

Movements of the foetus and polyhydramnion are not the only factors determining the length of the umbilical cord during pregnancy. The gender of the foetus, the mother’s pre-gravid weight and maternal weight gain also affect the length of the umbilical cord [28]. Female foetuses typically have shorter umbilical cords than male foetuses [17, 24, 26, 27]. Nevertheless, we observed no significant differences in foetal gender or maternal weight gain during pregnancy between the SSRI group and the control group. A few late pregnancy factors are also considered to modify umbilical cord length. Foetuses in breech presentation move less, which shortens the length of the umbilical cord [24]. However, the occurrence of breech presentation was similar between groups and therefore unlikely to have affected the results. Moreover, nulliparity might also result in shorter umbilical cords [26], and nulliparity was more common in the SSRI antidepressant group. However, adjustments for parity did not affect our findings.

A longer umbilical cord may reduce foetal circulation, wrap around the neck of the foetus and cause problems in the late period of pregnancy and labour. One retrospective study suggested that longer umbilical cords may associate with brain imaging abnormalities and/or abnormal neurological development in the offspring [29].

Our study had a reasonable sample size with 376 SSRI medicated mothers, and the data covered the entire population of labours between 2002 and 2012 in one university hospital. The use of antidepressant medication was reported by participants with a questionnaire and re-checked at admission to the hospital, adding to the reliability of the data concerning antidepressant use. Furthermore, we were also able to investigate the potential confounding effects of several maternal and pregnancy related factors on the observed associations.

Some limitations need to be taken into consideration while interpreting our findings. Firstly, due to a study setting that was based on the contents of an existing health register, we were unable to take into account the effect of diagnosed depression or depressive symptoms on the course of pregnancy and development of the foetus. This is a major weakness of the study, since the underlying depression itself may increase the risk of some pregnancy and delivery complications, such as preterm birth [30]. Nevertheless, we replicated many of the previously known observations that have been linked with SSRI use, and are not aware of any previous data suggesting that depression as such would lead to an increased umbilical cord length. Secondly, our data did not include information on the trimester-specific use of the SSRI medication or the administered dose of the medication. Such information could have helped to pinpoint the mechanisms underlying the observed changes, as the medication effects may be linked to certain developmental states of the foetus.
